# Alterations in Alanine Transaminase, Aspartate Transaminase, Gamma-Glutamyl Transpeptidase, and Creatine Kinase in Acne Patients Undergoing Isotretinoin Treatment: A Retrospective Evaluation of Laboratory Tests

**DOI:** 10.7759/cureus.57296

**Published:** 2024-03-30

**Authors:** Serap Maden

**Affiliations:** 1 Department of Dermatology, Near East University Faculty of Medicine, Nicosia, CYP

**Keywords:** gamma-glutamyl transpeptidase, creatine kinase, isotretinoin, aspartate transaminase, alanine transaminase, acne

## Abstract

Background

Isotretinoin therapy is a commonly prescribed medication by dermatologists for the treatment of acne. Regular laboratory assessments are recommended throughout the treatment period to detect any potential complications.

Objectives

This study aims to present the alterations in laboratory parameters throughout the course of isotretinoin therapy and identify required diagnostic testing.

Methods

This study involved 136 patients undergoing isotretinoin treatment at doses of 0.3-0.5 mg/kg/day, with ages ranging from 18 to 41 years. A retrospective evaluation was conducted on biomarkers including aspartate aminotransferase (AST), alanine aminotransferase (ALT), gamma-glutamyl transpeptidase (GGT), creatine kinase (CK), triglycerides, total cholesterol, low-density lipoprotein cholesterol (LDL-C), high-density lipoprotein cholesterol (HDL-C), hemoglobin, white blood cells, and thrombocytes. Levels of these parameters were analyzed prior to treatment and at the third month of treatment from the records of patients and the data were compared statistically. Moreover, the parameters of ALT, AST, CK, and GGT were graded objectively, and any alterations were noted in the patients.

Results

The levels of ALT, AST and GGT, along with triglycerides, total cholesterol, LDL-C, and thrombocyte levels showed significant elevation (p=0.001, p<0.001, p<0.001, p=0.001, p<0.001, p<0.001, and p=0.003, respectively). A significant decrease in HDL-C with hemoglobin was also noted (p=0.022, p=0.006, respectively). One patient (0.73%) exhibited grade 1 elevations in ALT, AST, and CK. One patient (0.73%) displayed grade 1 elevations in ALT and AST. One patient (0.73%) exhibited grade 1 elevations in AST and CK, while another patient (0.73%) had grade 1 elevation in AST and grade 3 elevation in CK. Furthermore, one patient (0.73%) had a grade 1 elevation exclusively in ALT, two patients (1.47%) had a grade 1 elevation exclusively in AST, and six patients (4.41%) exhibited a grade 1 elevation in CK only. No grade changes were observed in the GGT levels in the patients.

Conclusion

During isotretinoin treatment, changes in ALT and AST levels were more frequently associated with the muscle enzyme CK, while GGT levels remained unaffected. Therefore, GGT can be considered a reliable parameter for evaluating liver function in patients undergoing isotretinoin treatment.

## Introduction

Isotretinoin belongs to the first generation of drugs in the retinoid group and yields substantial improvement in acne within the first two to three months of use [1,2]. It is frequently prescribed as the first-line therapy for the treatment of severe nodulocystic and papulopustular acne, with the potential to cure the condition. It is also recommended for the management of moderate to severe acne [3]. Regular laboratory monitoring is recommended during the treatment period to identify any potential complications, such as hepatotoxicity, teratogenicity, rhabdomyolysis, leukopenia, thrombocytopenia, hyperlipidemia, and pancreatitis [4,5]. Laboratory tests during treatment with isotretinoin are becoming standardized, with recent research proposing a standard laboratory protocol for therapy [6]. However, selecting the appropriate tests for liver function is crucial. This is because gamma-glutamyl transpeptidase (GGT) has become more specific in denoting liver function than alanine aminotransferase (ALT) and aspartate aminotransferase (AST), which are correlated with creatine kinase (CK), a muscle enzyme [7]. The objective of this research is to demonstrate the changes observed in routine laboratory parameters, specifically ALT, AST, GGT, and CK, in patients receiving isotretinoin treatment, and evaluate their accordance with current literature recommendations.

## Materials and methods

This research was conducted as a retrospective study that analyzed data from acne patients who received isotretinoin treatment between March 2020 and March 2023. The study was approved by the Near East University Health Sciences Ethics Committee (2023/114 on 31/05/2023). The data was obtained from the electronic medical records at the Dermatology Clinic of Near East University Hospital, Cyprus. The study involved 136 patients aged 18 years or older who were diagnosed with severe papulopustular acne or moderate nodular acne and were treated with isotretinoin at a dosage of 0.3-0.5 mg/kg/day for at least three months. Before receiving treatment, all patients provided informed consent. Individuals with systemic conditions (such as liver, kidney, and hematological disorders), and a history of systemic drug use were excluded from the study. Blood samples were taken from patients before starting isotretinoin therapy and at one-month intervals during treatment for laboratory testing. In the study, patient data were collected, including demographic information and parameters such as ALT, AST, GGT, CK, total cholesterol, low-density lipoprotein cholesterol (LDL-C), high-density lipoprotein cholesterol (HDL-C), triglyceride, hemoglobin, white blood cells, and thrombocytes. The baseline laboratory levels of these parameters were compared to their levels after three months of treatment. Any abnormalities of ALT, AST, GGT, and CK were graded using the United States Department of Health and Human Services Common Terminology Criteria for Adverse Events (CTCAE) Version 5.0 grading system.

Statistical analysis

Continuous variables were reported as mean ± standard deviation and range, while count data was expressed as numerical values and percentages. The adequacy of the continuous variables with a normal distribution was assessed using the Kolmogorov-Smirnov test. For comparisons between the arithmetic means of continuous variables in different groups, the student’s t-test was employed for normally distributed groups, while the Mann-Whitney U-test was used for groups that did not conform to normal distribution. The paired-sample t-test was used within dependent groups that were suitable for normal distribution. The Wilcoxon signed-rank test was utilized to assess comparisons within dependent groups that did not adhere to the normal distribution. The data were recorded and analyzed using IBM SPSS Statistics for Windows, Version 25.0 (IBM Corp., Armonk, USA). The statistical significance level was p<0.05.

## Results

The study included 136 patients, with 95 (69.9%) being female and 41 (30.1%) being male. The age range of patients was predominantly 18 to 29 years old, with 129 patients (94.8%). Of the participants, 107 (78.7%) reported having an intermediate to high income, 131 (96.3%) reported having a college education, and 130 (95.6%) reported being single (Table 1). The levels of ALT, AST and GGT, along with triglycerides, total cholesterol, LDL-C, and thrombocyte levels showed significant elevation (p=0.001, p<0.001, p<0.001, p=0.001, p<0.001, p<0.001, and p=0.003, respectively). A significant decrease in HDL-C with hemoglobin was also noted (p=0.022, p=0.006, respectively). There was no statistically significant increase in CK levels, and the white blood cell count was reduced (p=0.105, p=0.491, respectively) (Table 2).

**Table 1:** Demographic data of the study participants The data has been presented using number (N) values and percentages (%).

VARIABLES		NUMBER (N)	PERCENTAGE (%)
GENDER	FEMALE	95	69.9
	MALE	41	30.1
AGE	18-29	129	94.8
	30-40	5	3.7
	≥41	2	1.5
SOCIOECONOMIC STATUS	LOW	29	21.3
	INTERMEDIATE/HIGH	107	78.7
EDUCATION	HIGH SCHOOL	5	3.7
	OVER COLLEGE	131	96.3
MARITAL STATUS	SINGLE	130	95.6
	MARRIED	6	4.4

**Table 2:** Laboratory data at baseline and after 3 months of isotretinoin treatment The data has been represented as Mean±SD. SD, standart deviation; ALT, alanine transaminase; AST, aspartate transaminase; GGT, gamma-glutamyl transpeptidase; CK, creatine kinase; TC, total cholesterol; LDL-C, low-density lipoprotein cholesterol; HDL-C, high -density lipoprotein cholesterol; WBC, white blood cell; HB, hemoglobin; U/L, units per liter; mg/dL; milligrams per deciliter, *significant at p<0.05.

LABORATORY PARAMETERS (REFERENCE RANGE)	BASELINE		3^RD^ MONTH		P VALUE
	MEAN±SD	RANGE	MEAN±SD	RANGE	
ALT (0-55 U/L)	18.86±9.64	7-68	22.36±13.96	7-105	0.001*
AST (5-34 U/L)	17.96±4.77	10-36	21.03±6.75	13-56	<0.001*
GGT (12-64 U/L)	16.96±8.26	7-55	19.30±9.24	4-63	<0.001*
CK (29-168 U/L)	98.82±90.51	21-733	101.19±92.30	32-857	0.105
TRIGLYCERIDE (<150 mg/dL)	87.15±42.05	11-283	98.88±45.96	43-289	0.001*
TC (<200 mg/dL)	174.96±28.88	108-262	187.41±31.13	117-276	<0.001*
LDL-C (<160mg/dL)	101.53±26.30	48-181	114.10±30.64	48-251	<0.001*
HDL-C (>60mg/dL)	56.20±11.67	31-96	54.77±12.68	31-104	0.022*
WBC (3.70-10.10 x103 U/L)	7.27±1.62	4.50-12.55	7.23±1.79	3.74-14.30	0.491
THROMBOCYTE (150-450x103 U/L)	271.96±62.42	134-431	281.64±64.24	142-471	0.003*
HB (10.80-14.20 mg/dL)	14.17±1.67	10.40-17.80	13.99±1.72	9.90-18.70	0.006*

**Figure 1:**
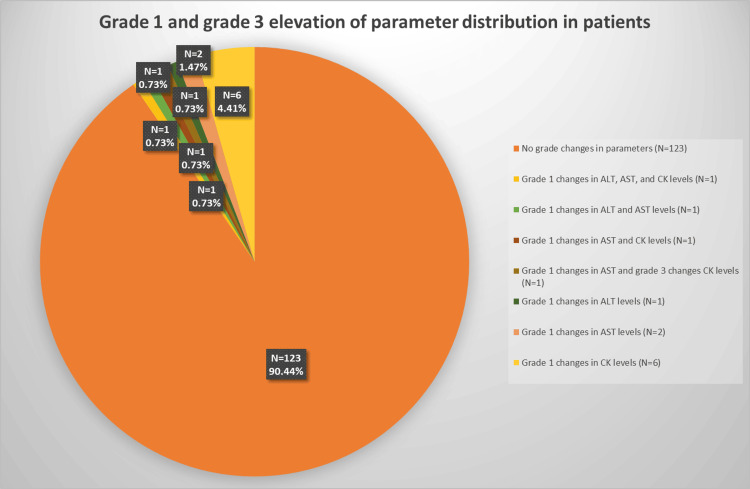
Grade alterations in parameters ALT, alanine transaminase; AST, aspartate transaminase; GGT, gamma-glutamyl transpeptidase; CK, creatine kinase. The parameters were evaluated using the United States Department of Health and Human Services Common Terminology Criteria for Adverse Events Version 5.0 grading system. The data has been presented using number (N) values and percentages (%).

The grading of increases in ALT, AST, CK, and GGT parameters followed the United States Department of Health and Human Services CTCAE version 5.0 grading system. Out of the 136 patients, 123 patients (90.44%) showed no changes in the grades of ALT, AST, GGT and CK parameters. One patient (0.73%) exhibited grade 1 elevations in ALT, AST, and CK, while GGT remained within the normal range. Another patient (0.73%) displayed grade 1 elevations in ALT and AST, but CK and GGT were within normal limits. One patient (0.73%) exhibited grade 1 elevations in AST and CK, while another patient (0.73%) had grade 1 elevation in AST and grade 3 elevation in CK. The patients’ ALT and GGT levels were within normal limits. Furthermore, one patient (0.73%) had a grade 1 elevation solely in ALT, while two patients (1.47%) had a grade 1 elevation solely in AST. Additionally, six patients (4.41%) displayed a grade 1 elevation in CK only (Figure 1).

## Discussion

Laboratory tests conducted during isotretinoin treatment have become increasingly standardized. Recent studies have contributed novel results to laboratory protocols for this therapy [6]. It was recommended to test GGT in addition to ALT and AST, as GGT is considered a more reliable indicator of liver injury. Furthermore, it was recommended checking CK levels at baseline and regularly monitoring physically active patients with baseline abnormalities and/or athletes engaging in intense physical activity [6].

In the third month after treatment with isotretinoin, our patients experienced a significant increase in ALT, AST, and GGT levels. Additionally, 13 patients exhibited changes in ALT, AST, and CK grades, depending on their grade classification. There were no changes in grade regarding GGT elevations. Webster et al. noted that GGT is more specific in denoting liver function than ALT and AST [7]. In addition to Webster et al. [7], another study also mentioned that extraneous factors, such as hemolysis or exercise, may contribute to elevated levels of ALT and AST, as GGT elevations were not observed alongside them [8]. In our study, we found that elevations in ALT and AST, as related to grading systems, were more relevant to CK than GGT. Apart from the liver, both ALT and AST are found throughout the body in skeletal muscle and the kidneys. In these tissues, AST is higher than ALT, and AST is also found in red blood cells [9]. In a case study, an increase in AST levels while undergoing isotretinoin treatment was found to be associated with an artefact in sample collection [10]. In conclusion, elevated levels of ALT and AST may result from sources outside the liver. Our investigation additionally supports the concept that GGT is a more reliable marker for liver function than both ALT and AST, as it remains comparatively steady under treatment, unlike ALT and AST. However, it is important to note that GGT is an enzyme that is found in hepatocytes and biliary epithelial cells, as well as the renal tubules, pancreas and intestine [9]. Additionally, certain incidents, such as alcohol ingestion or infection, can cause elevations in GGT regardless of isotretinoin [7]. 

In this study, it was not possible to associate elevated CK levels with exercise since the patients' exercise habits were not measured. However, it can be deduced from the fact that the patients are relatively young. Furthermore, the changes in the grade of CK were relevant in patients who had increases in ALT and AST where GGT showed no change in grade. Moreover, the increase in CK levels, alongside ALT and AST levels, can indicate that such elevations may originate from muscle tissue collectively. Similar to our study, Webster et al. [7] demonstrated that elevations in AST were commonly accompanied by increases in CK levels, implying a muscular origin of AST rather than liver-related. Moreover, ALT was less strongly associated with CK, but there was some intersection [7]. CK may be elevated by isotretinoin; however, it cannot be confirmed that isotretinoin is the sole cause, or if another factor such as exercise is contributing to the rise [11]. It is recommended that patients with CK levels approaching or surpassing five times the reference value should suspend or decrease physical activity or isotretinoin dosage until their CK level returns to the individual's pre-isotretinoin baseline level [11]. 

The increase in triglyceride, total cholesterol and LDL-C levels in our study cohort was within the acceptable range for a mild increase. Throughout the course of isotretinoin treatment, there was a low mean severity of cholesterol abnormalities that exceeded the maximum threshold. Specifically for triglycerides, it is recommended to repeat testing with fasting if their levels are elevated [6]. Identifying any increase in triglyceride levels over the course of treatment is crucial since it suggests a potential heightened risk of atherosclerosis in the future. Additionally, it is not advisable to continue using isotretinoin for extended periods in patients with underlying lipid disorders [12]. 

Throughout the treatment, hemoglobin and white blood cell count levels decreased, while thrombocyte cell counts increased. Additionally, the treatment did not result in any reported side effects. A meta-analysis showed that isotretinoin leads to significant alterations in the average values of certain laboratory tests (including white blood cell count and hepatic and lipid panels). However, these mean changes did not meet the predetermined criteria for high-risk, and only a small proportion of patients showed abnormalities in their laboratory results [13]. 

The study had some limitations, such as the absence of laboratory verifications at the end of isotretinoin therapy, failure to assess patients' use of extra herbal supplements (which are not in the medication category) that may affect liver enzymes, and lack of physical activity information. Furthermore, certain outcomes such as grade changes were seen in a limited number of patients.

## Conclusions

The results in this study suggest that GGT provides greater insight into the hepatic system than ALT and AST, while ALT and AST are associated with CK changes. To evaluate hepatotoxicity prior to and during isotretinoin treatment, this study recommends assessing GGT along with ALT and AST. Additionally, for athletes, CK should be evaluated to detect muscle abnormalities. During ongoing treatment, the timing intervals for testing may depend on the test results and risk factors of the patients such as hepatotoxicity or excessive exercise. 
